# Auxin-Mediated Regulation of Dorsal Vascular Cell Development May Be Responsible for Sucrose Phloem Unloading in Large Panicle Rice

**DOI:** 10.3389/fpls.2021.630997

**Published:** 2021-02-25

**Authors:** Yao Deng, Yongchao Yu, Yuxiang Hu, Li Ma, Yan Lin, Yue Wu, Zhen Wang, Ziteng Wang, Jiaqi Bai, Yanfeng Ding, Lin Chen

**Affiliations:** ^1^College of Agriculture, Nanjing Agricultural University, Nanjing, China; ^2^Key Laboratory of Crop Physiology and Ecology in Southern China, Ministry of Agricultural University, Nanjing, China; ^3^Jiangsu Collaborative Innovation Center for Modern Crop Production, Nanjing, China

**Keywords:** rice, grain filling, auxin, vascular cell development, sucrose phloem unloading, inferior spikelets

## Abstract

Large panicle rice cultivars often fail to fulfill their high-yield potential due to the poor grain filling of inferior spikelets (IS), which appears as initially stagnant development and low final seed weight. Understanding the mechanism of the initial stagnancy is important to improve IS grain filling. In this study, superior spikelets (SS) were removed from two homozygous japonica rice varieties (W1844 and CJ03) with the same sink capacity in an attempt to force photosynthate transport to the IS. The results showed that SS removal increased the grain weight, sucrose content, starch accumulation, and endogenous IAA levels of IS during the initial grain-filling stage. SS removal also improved the patterns of vascular cells in the dorsal pericarp and the expression levels of genes involved in sucrose transport (*OsSUTs* and *OsSWEETs*) and IAA metabolism (*OsYUCs* and *OsPINs*). Exogenous IAA application advanced the initiation of grain filling by increasing the sucrose content and the gene expression levels of sucrose transporters. These results indicate that auxin may act like a signal substance and play a vital role in initial grain filling by regulating dorsal vascular cell development and sucrose phloem unloading into caryopsis.

## Introduction

Rice (*Oryza sativa* L.) is the main food source for more than 50% of the world’s population. Sink capacity and grain-filling efficiency can finally determine the rice yield (Kato and Takeda, 1996; Fageria, 2007). Large panicle rice varieties with high sink capacity that exhibit numerous spikelets per panicle are widely used in modern production (Cheng et al., 2007). However, this type never reaches the theoretical maximum yield due to the poor grain-filling quality (Ao et al., 2008; Yang and Zhang, 2010). The grain-filling rate and grain size have a close relationship with the spikelet location on panicles. Generally, the superior spikelets (SS) are located on the apical primary branches, flowering earlier and filling fast, while the inferior spikelets (IS) are located on proximal lower secondary branches, flowering later and filling slowly (Mohapatra et al., 1993; Ishimaru et al., 2003). The poor inferior spikelet filling is a limiting factor to further enhance grain yield potential, and it is often related to the insufficient supply and utilization of assimilates (Murty and Murty, 1982; Ishimaru et al., 2005). Although much work has been done to explain the relationship between carbohydrate accumulation and spikelet development (Zhu et al., 1988; Mohapatra and Sahu, 1991; Mohapatra et al., 1993, 2000), no clear mechanism can underly the poor grain filling.

Leaf photosynthesis and reserved non-structural carbohydrate (NSC) redistribution in the stem and sheath can determine the grain filling quality and final yield (Cock and Yoshida, 1972; Tsukaguchi et al., 2008). Sucrose is the main solute transported from the source to the sink via the phloem (Braun, 2012; Braun et al., 2014). It has been proposed that sucrose is first unloaded from the phloem at the dorsal vascular bundle to enter into the pericarp, then the sugars will be transferred into the developing caryopsis to supply nutrients for endosperm development (Oparka and Gates, 1984; Zhang et al., 2007; Wu et al., 2016). *CRR1* encodes a protein homologous to the Arabidopsis callose synthases AtGSL8 and AtGSL10. The loss function of CRR1 will retard ovary expansion. In rice, the *crr1* mutant, which exhibits disordered patterns of dorsal vascular cells in the ovary, showed defective caryopsis development and poor grain filling (Song et al., 2016). Vascular tissues are terminated in maternal tissues, which are symplasmically isolated from filial tissues (Oparka and Gates, 1981), thus, transporters are essential for sugar release from maternal tissues and subsequent reuptake into filial tissues. Recent studies have shown that two classes of plasma membrane sucrose transporters, SUTs (Sucrose Transporters) and SWEETs (Sugars Will Eventually be Exported Transporters), take on key roles in sucrose transmembrane transport from maternal tissues to filial tissues (Aoki et al., 2003; Chen et al., 2012). The loss function of genes encoding these transporters always resulted in defects in endosperm development and seed filling (Scofield et al., 2002; Ma et al., 2017; Yang et al., 2018). Five SUT coding genes (*OsSUT1, OsSUT2, OsSUT3, OsSUT4*, and *OsSUT5*) have been identified in rice (Aoki et al., 2003). Their expression character showed different during the grain-filling stage. The expression level of *OsSUT1* reached a peak at 7 DPA (days post-anthesis) and decreased after that. The expression levels of *OsSUT2*, *OsSUT4*, and *OsSUT5* showed a similar pattern, peaking at 2–4 DPA. The loss function of OsSUT1 resulted in poor grain filling and lower seed setting rate, indicating the important role of OsSUT1 taking in sucrose unloading in a spikelet (Scofield et al., 2002). Overexpression of *OsSUT2* and *OsSUT5* can also enhance the sucrose unloading, but the detailed reason remains unknown. In the early grain filling stage, the *OsSUT1* was expressed in dorsal vascular bundles, nucellar projection, and dorsal aleurone (Furbank et al., 2001; Ishimaru et al., 2001). *OsSUT4* was expressed in the aleurone and embryo, and the *OsSUT3* was expressed in dorsal aleurone (Chung et al., 2014; Bai et al., 2016). All the above demonstrate the important roles of OsSUT1/3/4 playing in the sucrose transport from dorsal phloem to filial aleurone. SWEETs, a family of membrane proteins, mediate sugar efflux or influx (Chen et al., 2010). AtSweet11 and AtSweet12 played a key role in sucrose efflux from parenchyma to the apoplastic before sucrose loaded into phloem in Arabidopsis (Chen et al., 2012). Further, they reported AtSweet11, together with AtSweet12 and AtSweet15, were involved in seed development of Arabidopsis (Chen et al., 2015). As a homolog of *AtSweet11*, the *OsSweet11* of rice is expressed in developing rice caryopsis, and it is an important sugar transporter during the early stage of rice grain filling (Ma et al., 2017). Yang also reported that SWEET11 and SWEET15 were key players in rice grain filling, involved in sucrose efflux at nucellar projection and transferring across the aleurone interface (Yang et al., 2018). Mutants of both maize *ZmSWEET4c* and its rice ortholog *OsSWEET4* showed defective in seed filling, indicating that a lack of hexose transport at the basal endosperm transfer layer impairs the further transfer of sugars imported from the maternal phloem. In both maize and rice, SWEET4 was likely recruited to enhance sugar import into the endosperm (Sosso et al., 2015). However, to our knowledge, the difference between SS and IS filling has scarcely been studied in relation to the development of dorsal vascular bundles and the comprehensive expression patterns of the sugar transporters.

Auxin is a critical plant growth regulator that modulates diverse physiological processes, such as tropic responses to light and gravity, general root and shoot architecture, organ formation, vascular patterning, and tissue development (Davies, 1995; Reinhardt, 2003; Kepinski and Leyser, 2005; Overvoorde et al., 2010). It can also induce the development, differentiation, patterning of vascular cells, and the permeability of plasmodesmata linked with cells by its polar transport (Berleth et al., 2000; Scarpella et al., 2010; Han et al., 2014), which determines the translocation efficiency of nutrients. The rates of endosperm cell division and grain filling were closely associated with IAA contents in grains (Zhang et al., 2009). Much lower IAA existed in IS than SS at the early grain-filling period (You et al., 2016), suggesting that auxin may be the key determinant of asynchronous grain filling between SS and IS. Auxin also promoted the translocation of sucrose to developing grains, the accumulation of starch, and the activity of related metabolic enzymes in grain (Cole and Patrick, 1998; Javid et al., 2011). However, at present, there is still no clear evidence underlying IAA involvement in grain filling.

In this study, we observed the changes in initial dorsal vascular cell development, sucrose transport, and IAA metabolism in the developing grain of IS to understand the role of auxin in grain filling. We provided evidence that the different IAA levels of SS and IS mediated the development of dorsal vascular cells and affected the sucrose phloem unloading efficiency in rice grain filling.

## Materials and Methods

### Plant Materials and Growth Conditions

Field experiments were performed during rice growing seasons in 2018 at Danyang Experimental Station of Nanjing Agricultural University, Jiangsu Province, China (31°54′31″N, 119°28′21″E). Two homozygous large panicle japonica rice strains, W1844 and CJ03, from the State Key Laboratory of Rice Genetics and Germplasm Innovation, Nanjing Agricultural University were used. The agronomic traits are shown in Table 1. Seedlings were field-grown and transplanted 22 days after sowing (May 21) at a hill spacing of 13.3 cm × 30 cm with three seedlings per hill. The individual plot size was 50 m^2^, and the plots were arranged in a randomized block design with three replicates for each treatment. The soil at the experimental site was clay loam. The amount of nitrogen application was 240 kg ha^–1^. The application ratio of base fertilizer to panicle fertilizer was 5:5. Base fertilizer and panicle fertilizer were applied before transplanting and the 3.5 leaf-age remainders, respectively. The heading date of W1844 and CJ03 was on August 21–23.

**TABLE 1:** The agronomic traits of CJ03 and W1844.

Material	Plant height (cm)	Panicle length (cm)	Grains per panicle	Grain growth density	Setting rate	1,000-grain weight (g)
CJ03	100.41a	21.63a	277.10a	12.81a	0.92a	22.56b
W1844	99.27a	20.80a	245.07a	11.78a	0.90a	26.50a

*Different lowercase letters following the data in the same column under the same line indicates significant differences at P < 0.05 among materials.*

### Sampling and Treatment

Spikelets were classified equally into three parts by their position within a panicle: upper, middle, and lower parts (You et al., 2016). The grains on the three top primary branches at the upper part are superior spikelets (SS), The grains on the three bottom secondary branches at the lower part are inferior spikelets (IS). Spikelet thinning treatments were performed on the panicles tagged at the flowering date of inferior spikelets in three groups: control group (labeled CK); the upper 1/3 of spikelets removed group (R1);, and the upper 2/3 of the spikelets removed group (R2).

To study the effect of auxin on grain filling individually, an IAA (indole-3-acetic acid, Sigma) solution was sprayed on the leaves and panicles during the initial grain-filling phase (labeled IAA), which started the flowering date of superior spikelets. The concentration of IAA was 20 mg/L, which was based on earlier reports of IAA application (Xu et al., 2007; Zhang et al., 2009). Exogenous IAA was sprayed separately at 150 mL/m^2^ per plot in the evening (after sunset) daily for 3 days.

At the individual flowering date of superior spikelets and inferior spikelets, 300 (for superior spikelets) and 500 (for inferior spikelets) panicles with similar growth patterns were tagged separately for each plot. We sampled 45 (for superior spikelets) and 75 (for inferior spikelets) panicles from each plot every 2 days from anthesis to 12 days post-anthesis (DPA). The SS and IS were collected from the CK and IAA groups, and the IS were collected from the R1 and R2 groups. Three of the 5 sampled grains were frozen in liquid nitrogen for 1 min before storing at −80°C for the determination of IAA and gene expression levels. The remaining grains were deactivated at 105°C for 0.5 h and dried at 80°C to a constant weight to determine grain weight, sucrose content, and starch content. At maturity, approximately 250 tagged complete panicles with no grain loss from each treatment were harvested for the measurement of yield components.

### Yield Components

The spikelets of panicles collected at maturity and the grains were dried to a constant weight at 80°C. The grains were weighed and dehulled to determine the grain dry weight (DW). The 1,000-grain weight and seed-setting rate were individually calculated. The seed-setting rate was calculated using the method: plump grain number/total grain number (Kobata et al., 2013).

### Grain Weight and Grain Growth Rate

Richards’s growth equation was used to fit the grain-filling processes (Richards, 1959).

(1) $$\text{W}=\frac{A}{{\left(1+Be^{-kt}\right)}^{1/N}}$$

The grain-filling rate (R) was calculated as the derivative of Eq. 2

(2) $$\text{R}=\frac{AkBe^{-kt}}{N{\left(1+Be^{-kt}\right)}^{\left(N+1\right)/N}}$$

W, grain weight (mg); A, final grain weight (mg); t, time after anthesis (days);B, k, and N, coefficients established from the regression equation.

### Sucrose and Starch Content

The determination of sucrose and starch content was referred to Yoshida’s study (Yoshida, 1972). The collected spikelets were dried at 80°C to a constant weight and ground to powder. 0.1 g of the sample was extracted by 80% aqueous ethanol at 80°C for 30 min. Then the sample was cooled and centrifuged at 5,000 rpm for 15 min. The supernatant was collected with a 10 mL volumetric flask. All the supernatants were combined in the flask with the addition of distilled water to 10 mL. The extract was filtered through a 0.45 μm Millipore membrane, and UPLC-ELSD was used to filter the extract and analyze the sucrose. The following was the conditions used for the UPLC system (UltiMate^TM^ 3000, Thermo Fisher Scientific^TM^): index detector, ELSD 6000 (Agilent); column, Shoedx sugar column SC1011; column temperature, 30°C; mobile phase, a solvent mixture of acetonitrile and ultra-pure water (75:25 v/v); flow rate, 1.0 mL/min; and injection volume, 20 μL.

The residue after centrifugation in the tube was used to determine the starch content. Firstly, it was oven-dried at 60°C, then, 2 mL of distilled water was added, and it was boiled in a water bath for 20 min. After cooling, 2 ml of 9.2 mol L^–1^ HClO_4_ was added and vortexed for 10 min. After that, the sample was centrifuged at 5,000 rpm for 15 min, the supernatant was then collected in a 50 mL volumetric flask, and the residue was soaked in HClO_4_. All the supernatants were combined in the flask, and distilled water was added up to 50 mL. The anthrone method was used to determine the starch content. The extract (0.1 mL) and 4 mL of 0.2% anthrone were added to a new 15 mL centrifuge tube and then given an 80°C water bath for 15 min. Colorimetric determination was performed using a chromometer at OD 620 nm.

### Microscopic Analysis of Dorsal Vascular Cells

Developing grains at 4 and 10 DAP were collected, fixed in 2.5% glutaraldehyde in 0.1 M phosphate-buffered saline (pH 7.2) at 4°C for 24 h and then post-fixed in 1% OsO_4_ buffer at room temperature for 12 h. Samples were sequentially dehydrated through an acetone series, embedded in epoxy resin, penetrated under 37°C overnight, and polymerized under a gradual temperature increase (up to 60°C) for 1.5 days. Ultrathin sections (50–60 nm) were cut using an ultramicrotome (LKB) and contrasted with uranyl acetate/lead citrate for transmission electron microscope (TEM, H-7650, HITACHI).

### Endogenous IAA Content

IAA was determined by the method modified from Yang et al. (2001) and Choudhary et al. (2012). 0.5 g dehulled grains were ground in liquid nitrogen into a fine powder. Samples were homogenized in 2 mL 80% methanol extraction buffer, kept at 4°C for 12 h, and centrifuged at 5,000 rpm for 15 min at the same temperature. All the supernatants were combined in a 10 mL tube and then purified further using poly-vinylpolypyrrolidone (PVPP, Solarbio) and a Sep-Pak C18 cartridge (Waters) with a methanol gradient elution. Eluants were dried in a freeze-dryer (Scanvac), dissolved in 500 μL 50% (v/v) methanol, and filtered through a 0.22 μm Millipore membrane. The purified product was subjected to ultra-performance liquid chromatography (UPLC, ACQUITY UPLC H-Class system, Waters) analysis. UPLC analysis was performed using an ACQUITY UPLC HSS T_3_ column (100 mm × 2.1 mm × 1.8 μm, Waters). The mobile phase A solvent consisted of methanol, and the mobile phase B solvent consisted of 0.1% acetic acid. The injection volume was 2 μL, and the flow rate was adjusted to 0.3 mL/min. The wavelength used for IAA detection was 230 nm. Pure IAA (Sigma), dissolved in HPLC grade methanol, was used as the standard for identification and quantification.

### Quantitative Real-Time RT-PCR Analysis

The transcriptional analysis of the related genes, which were selected according to previous research (Aoki et al., 2003; Lim et al., 2006; Abu-Zaitoon et al., 2012; Sosso et al., 2015; Bai et al., 2016; Sharma et al., 2018; Yang et al., 2018). An RNA extraction kit (DP432, TIANGEN) was used to isolate the total RNA from the dehulled grains. Total RNA was reversed-transcribed into first-strand cDNA using a PrimeScript^TM^ RT Reagent Kit (Code No. RR037A, Takara) and oligo-dT. We conducted qRT-PCR using SYBR Premix Ex Taq^TM^ (Code No. RR420A, Takara) according to the manufacturer’s protocol by ABI 7300 sequencer. The relative expression levels of all of genes were determined based on the 2^−ΔΔCT^ method (Livak and Schmittgen, 2001). *Actin* was the reference gene, and the gene expression levels in SS at 4 DAP under CK treatment were set as the control. The primers used are listed in Table 2.

**TABLE 2:** Sequence of primers for Actin and related Genes used for qRT-PCR.

Gene name	Locus	Forward primer	Reverse primer
*Actin*	LOC_Os03g50885	CAATCGTGAGAAGATGACCC	GTCCATCAGGAAGCTCGTAGC
*OsSUT1*	LOC_Os03g07480	GCTTTCAACCAGGGTGTCAG	ACTTTCCGGCACATTGGTTC
*OsSUT3*	LOC_Os10g26470	TTTGGAAATGTTAGGCGCCC	ATAGCCTATCAGCCGTCCAC
*OsSUT4*	LOC_Os02g58080	CTCGTGCCCTTTTAGCTGAC	AACGTTTCCAACAGCCATCC
*OsSWEET4*	LOC_Os02g19820	TTTAGCTAAGCCGTCCCAAAG	CACGGCATGATGATGATATTG
*OsSWEET11*	LOC_Os08g42350	GGGATTTCTGGCTAGTTTCT	CGAGGTAGAGGACGATGTAG
*OsSWEET15*	LOC_Os02g30910	CTTCACCTTTGGCATCTTAG	AACGCGTAGTACATCCACA
*OsYUC9*	LOC_Os01g16714	ACCTCCATCGTCATCCGCAG	ACATGAGCAGCACCACCTTG
*OsYUC11*	LOC_Os12g08780	TAAGGCAAATACTGGTCGGT	GTGTAGCCTGTAGCAAAAAC
*OsTAR1*	LOC_Os05g07720	GCACCATACTACTCCTCGTACCC	GACGAGCTCGACGTAGGTGT
*OsPIN1b*	LOC_Os02g50960	GAACACCTACTCCAGCCTCATCG	GCCATGAACAGACCGAGACTGAAC
*OsPIN5b*	LOC_Os08g41720	CGATTGCTTCCTTCGTTTTCGCAAAGG	CAGAATCGGCAGAGAGATCAATGTTCC
*OsPIN10a*	LOC_Os01g45550	TCATCCGCAACCCCAACACTTAC	ATTTGCCACACGCGATGATGCTG

### Statistical Analysis

Statistical analyses of the data were performed using Microsoft Excel 2016, SPSS 25.0, and Origin 2017. One-way analysis of variance was performed, and the least significant difference test (LSD test) at a 5% probability level was used for estimations of significant differences of the means of investigated traits.

## Results

### Grain Weight and Seed-Setting Rate

Compared with IS, the SS of W1844 and CJ03 exhibited the highest grain weight and seed-setting rate (Table 3). After spikelet thinning, the grain weights and seed-setting rates of IS were significantly increased, and the values of the R2 treatment were higher than those of the R1 treatment. For W1844, the grain weight and seed setting of IS under the R2 treatment improved considerably and even approached the values of SS in the CK group. Therefore, we focused on the effect of the R2 treatment on IS grain filling. However, the grain weight of IS under the R2 treatment for CJ03 remained lower than that of the SS in the CK group. Overall, spikelet thinning treatments significantly improved the grain weight and seed-setting rate of IS.

**TABLE 3:** Grain weight and seed setting rate of CJ03 and W1844 under spikelets thinning treatments.

Material	Treatment	Grain weight (mg/grain)		Seed settting (%)	
		Superior	Inferior	Superior	Inferior
CJ03	CK	25.72a	19.93c	95.44a	82.90b
	R1	−	21.04b	−	84.78b
	R2	−	21.11b	−	92.68a
W1844	CK	27.68a	22.41c	91.38a	81.25b
	R1	−	24.94b	−	85.09b
	R2	−	26.06a	−	91.04a

*CK, control group; R1, upper part of the spikelets were removed; R2, upper and middle parts of the spikelets were removd; -, the spikelets that were removed. Different lowercase letters following the data that were belong to the same character and variety indicates significant differences at P < 0.05.*

### Grain Weights of Superior and Inferior Spikelets at the Initial Grain-Filling Stage

**FIGURE 1:**
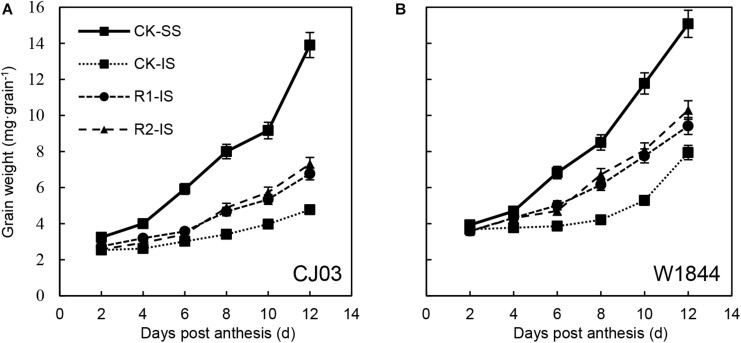
Grain weight **(A,B)** of SS and IS of CJ03 and W1844 at the early grain-filling stage. CK, control group; R1, upper 1/3 part of the spikelets were removed; R2, upper 2/3 part of the spikelets were removed. CK-SS, superior spikelets of control panicle, CK-IS, inferior spikelets of control panicle, R1-IS, inferior spikelets of upper 1/3 removed panicle, R2-IS, inferior spikelets of upper 2/3 removed panicle. Vertical bars represent the mean values ± SE (*n* = 3).

The changes in grain weight of IS treated with the two materials during the initial grain-filling stage were quite different. The grain weight of IS started increasing rapidly at 8–10 DAP in W1844, while the grain weight of IS increased slowly until 12 DAP in CJ03 (Figure 1). This result indicates that the IS of CJ03 have a longer lag period than those of W1844. After spikelet thinning, the progress of initial grain filling was advanced. For W1844, the grain weight of IS started increasing at 4–6 DAP, while the IS of CJ03 started filling at 8 DAP. However, for neither W1844 nor CJ03, the initial grain filling of IS in the R2 treatment group reached levels similar to that of SS in the CK group.

### Carbohydrate Contents in Developing Grains

**FIGURE 2:**
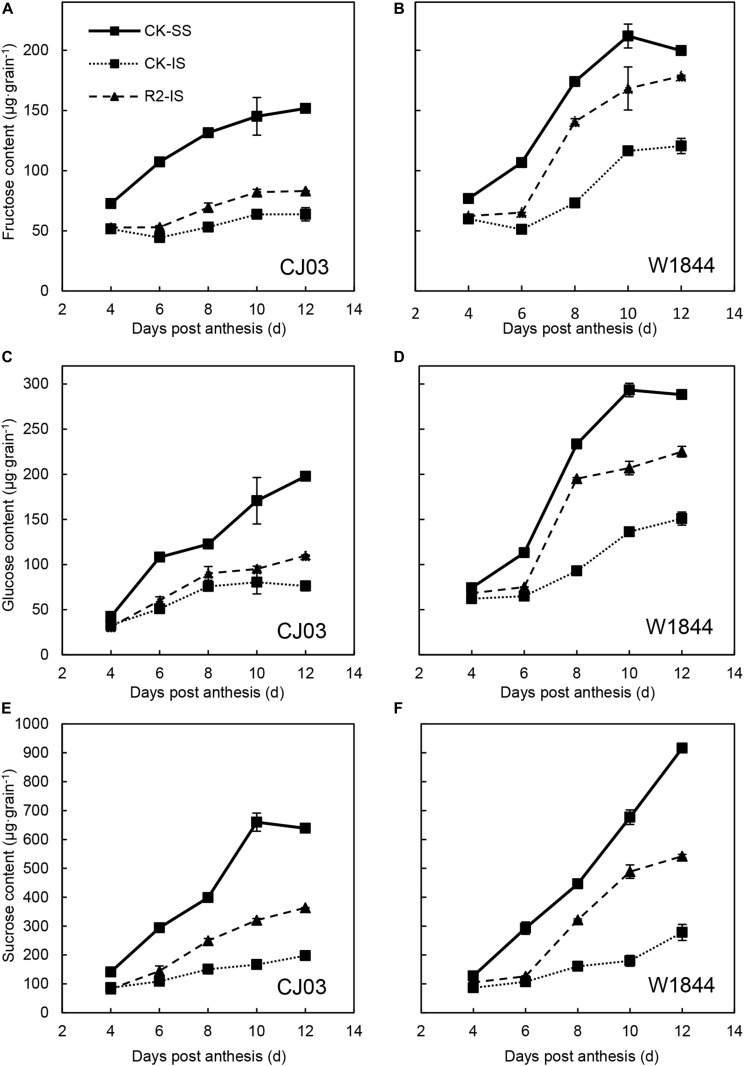
Fructose **(A,B)**, glucose **(C,D)**, and sucrose **(E,F)** contents of SS and IS of CJ03 and W1844 at the early grain filling stage. CK-SS, superior spikelets of control panicle, CK-IS, inferior spikelets of control panicle, R2-IS, inferior spikelets of upper 2/3 removed panicle. Vertical bars represent the mean values ± SE (*n* = 3).

Figure 2 illustrates the changes in the fructose, glucose, and sucrose content of the SS and IS of CJ03 and W1844 during the early grain-filling period. The sugar content of SS was significantly higher than those of IS and kept increasing rapidly. In contrast, the sugar content of IS increased slowly. The sugar contents in both SS and IS of W1844 were higher than those of CJ03. After SS was removed, the sugar contents of IS started increasing rapidly from 8 DAP compared to those of IS in the CK group. The sugar contents of IS in the R2 treatment group were much closer to those of the SS in the CK group of W1844 compared to CJ03. Briefly, the changes in IS carbohydrate contents increased significantly after the SS were removed, which suggests that the IS obtained sufficient carbohydrates after the SS were removed.

### Dorsal Vascular Cell Development in Developing Grains

**FIGURE 3:**
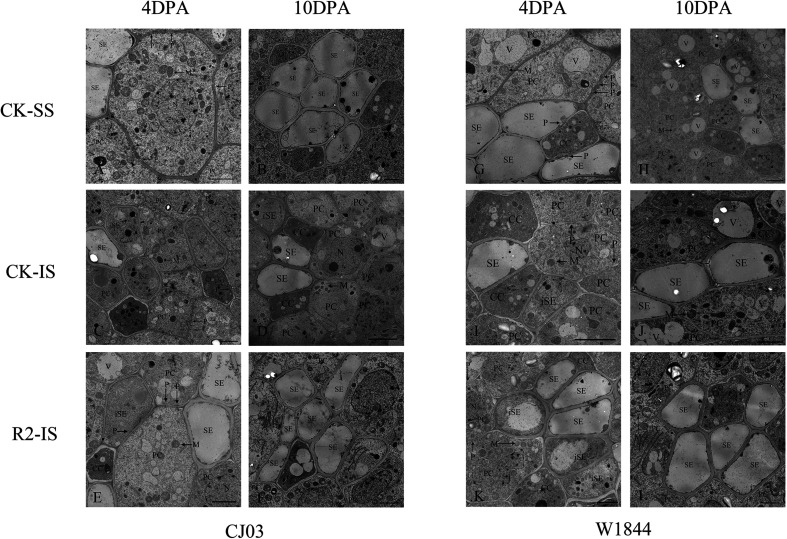
Ultrastructure of the dorsal phloem of the vascular bundle in CJ03 and W1844 caryopsis at the early grain-filling stage. A, G, Ultrastructure of the dorsal vascular bundle at 4 days post-anthesis in superior spikelets; B, H, Ultrastructure of the dorsal vascular bundle at 10 days post-anthesis in superior spikelets; C, I, Ultrastructure of the dorsal vascular bundle at 4 days post-anthesis in inferior spikelets; D, J, Ultrastructure of the dorsal vascular bundle at 10 days post-anthesis in inferior spikelets; E, K, Ultrastructure of the dorsal vascular bundle at 4 days post-anthesis in inferior spikelets under spikelet removal; F, L, Ultrastructure of the dorsal vascular bundle at 10 days post-anthesis in inferior spikelets under spikelet removal. SE, sieve element; iSE, immature sieve element; CC, companion cell; PC, parenchyma cells; P, plasmodesmata; M, mitochondrion; N, Nucleus. Bar = 2 μm.

To investigate the differences in the sucrose phloem unloading ability between SS and IS during initial grain filling, the ultrastructure of phloem cells in the dorsal vascular bundles from developing caryopses collected at 4 DPA and 10 DPA were observed by TEM (Figure 3). The phloem in the pericarp bundle consists of sieve elements (SEs), companion cells (CCs), and parenchyma (Oparka and Gates, 1981). SEs were associated with CCs, surrounded by parenchyma, and connected to these vascular elements via plasmodesmata. At 4 DAP, SEs in the pericarp of the SS were well developed with parenchyma filled with organelles, such as mitochondria, and connected other vascular cells with abundant plasmodesmata (Figure 3A). In contrast, most SEs in the pericarp of IS remained immature at the same time and contained cytoplasm (Figure 3C). The plasmodesmata were also rarely found between contiguous tissues of vascular bundles in the 4 DAP IS. Until 10 DAP, most phloem cells in the pericarp of the IS became structurally mature, with occasional immature sieve elements (Figure 3D). After the SS were removed, the development of phloem cells soon improved. At 4 DAP, several SEs in the pericarp of the IS subjected to the R2 treatment had a mature structure, while immature SEs were removing their inclusions rapidly (Figure 3E). Compared with CJ03, the development of phloem cells in the IS started much earlier in W1844, which was improved and more efficient by spikelet thinning. This result indicates that improvements in IS grain filling may be related to the development of phloem cells in the pericarp.

### Expression Levels of Genes Involved in Sucrose Phloem Unloading and Transport in Developing Grains

**FIGURE 4:**
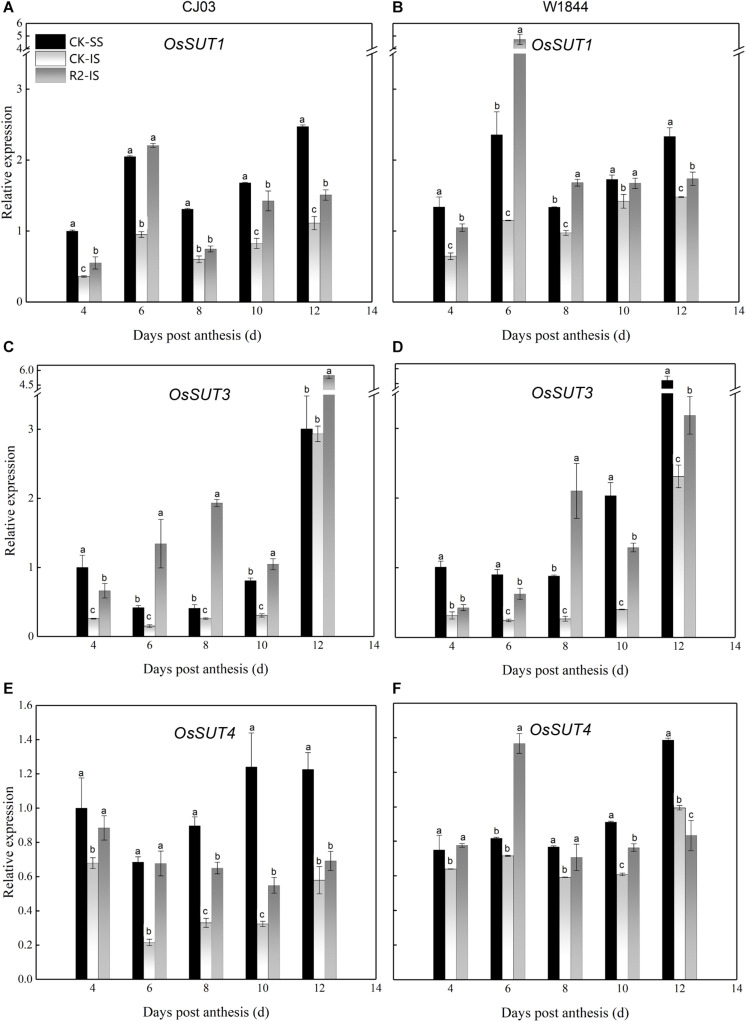
Relative expression levels of *OsSUT1* **(A,B)**, *OsSUT3* **(C,D)**, and *OsSUT4* **(E,F)** in SS and IS at the early grain-filling stage of CJ03 and W1844. CK-SS, superior spikelets of control panicle, CK-IS, inferior spikelets of control panicle, R2-IS, inferior spikelets of upper 2/3 removed panicle. Vertical bars represent the mean values ± SE (*n* = 3). Significant differences at each time point are indicated by different letters (*P* < 0.05) as determined by Duncan’s test.

**FIGURE 5:**
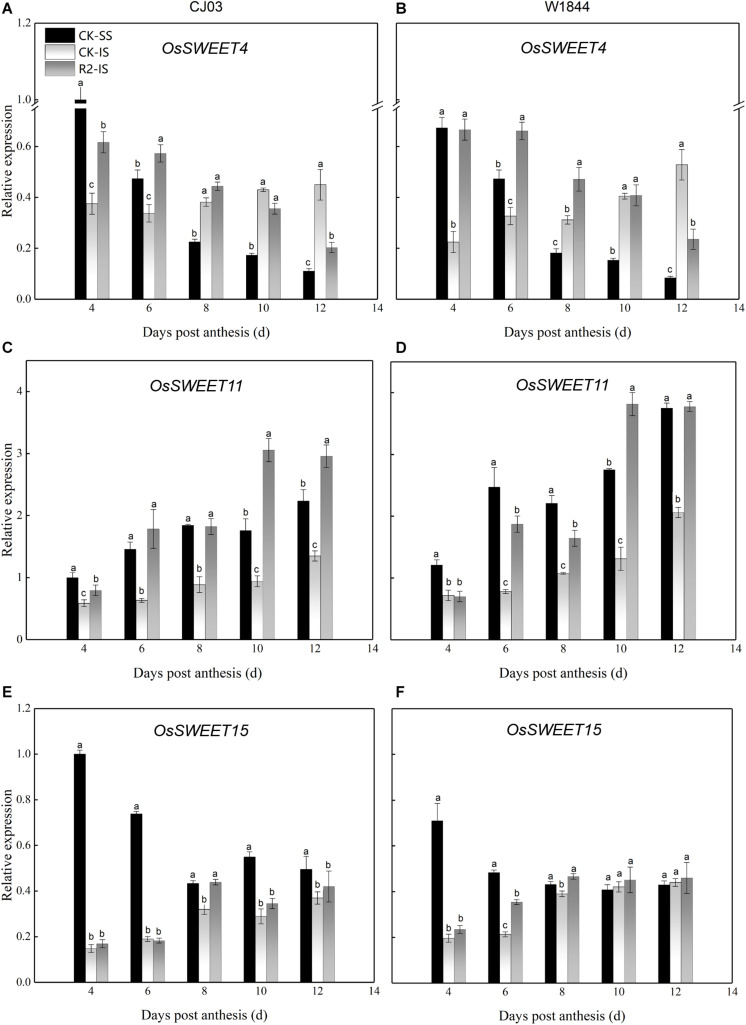
Relative expression levels of *OsSWEET4* **(A,B)**, *OsSWEET11* **(C,D)**, and *OsSWEET15* **(E,F)** in SS and IS at the early grain-filling stage of CJ03 and W1844. CK-SS, superior spikelets of control panicle, CK-IS, inferior spikelets of control panicle, R2-IS, inferior spikelets of upper 2/3 removed panicle. Vertical bars represent the mean values ± SE (*n* = 3). Significant differences at each time point are indicated by different letters (*P* < 0.05) as determined by Duncan’s test.

To determine the differences in sucrose transport ability, we measured the relative expression levels of *OsSUTs* and *OsSWEETs* in the developing grains (Figures 4, 5). The results showed different expression patterns of *OsSUT1*, *OsSUT3*, *OsSUT4*, *OsSWEET4*, *OsSWEET11*, and *OsSWEET15* at the initial filing stage, which appeared to be similar between varieties. Compared with IS, the expression levels of *OsSUT1*, *OsSUT3*, *OsSUT4*, and *OsSWEET11* in SS were relatively higher during the early grain-filling stage, while the expression levels of *OsSWEET4 and OsSWEET15* in SS were only higher shortly after anthesis. After SS removal, the expression levels of *OsSUT1*, *OsSUT3*, *OsSUT4*, and *OsSWEET11* in the IS increased, and the expression levels of *OsSWEET4* and *OsSWEET15* in the IS were changed to patterns more similar to the SS in the CK group. Notably, the expression levels of these sucrose transporter genes in the IS subjected to the R2 treatment increased significantly at 6 DAP compared to those in the IS in the CK group, especially the expression levels of *OsSUT1*, *OsSUT4*, and *OsSWEET4* in W1844. These results show that more carbohydrates were transported into the endosperm and used for growth after the improvement of vascular development.

### Starch Content in Developing Grains

**FIGURE 6:**
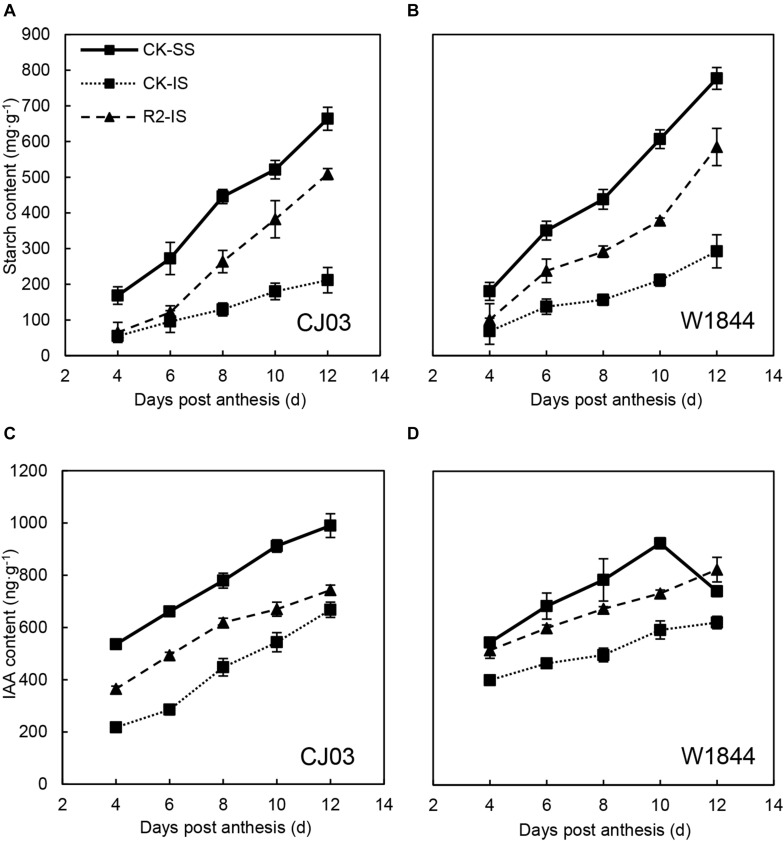
Starch **(A,B)** and IAA **(C,D)** contents of SS and IS of rice at the early grain-filling stage of CJ03 and W1844. CK-SS, superior spikelets of control panicle, CK-IS, inferior spikelets of control panicle, R2-IS, inferior spikelets of upper 2/3 removed panicle. Vertical bars represent the mean values ± SE (*n* = 3).

Figure 6 illustrates the starch contents of SS and IS from CJ03 and W1844 during the initial grain-filling period. The changes in starch content were similar to the changes in grain weight. The starch content of SS was significantly higher than that of IS and kept increasing rapidly. In contrast, the starch content of IS increased slowly compared to that of SS. Furthermore, the starch content in the IS of W1844 was higher than that of CJ03, while the starch content of the SS was similar between varieties. After the SS were removed, the starch content of the IS in CJ03 started increasing rapidly from 8 DAP compared to that of IS in the CK group, while for W1844, the rapid increase began from 6 DAP. Briefly, the IS starch content increased significantly after the SS were removed.

### IAA Content and Metabolism in Developing Grains

As shown in Figure 6, the IAA content of SS was significantly higher than that of IS during the initial grain-filling stage. Compared with CJ03, the highest IAA content in the SS of W1844 occurred earlier, at 10 DAP. After SS removal, the IAA content of IS increased sharply, with the IAA content in the IS of W1844 reaching a level beyond that of the SS at 12 DAP. This result indicates that increasing the IAA levels improved IS grain filling. In CJ03, the IAA content of IS after SS removal increased but was still much lower than that of SS.

**FIGURE 7:**
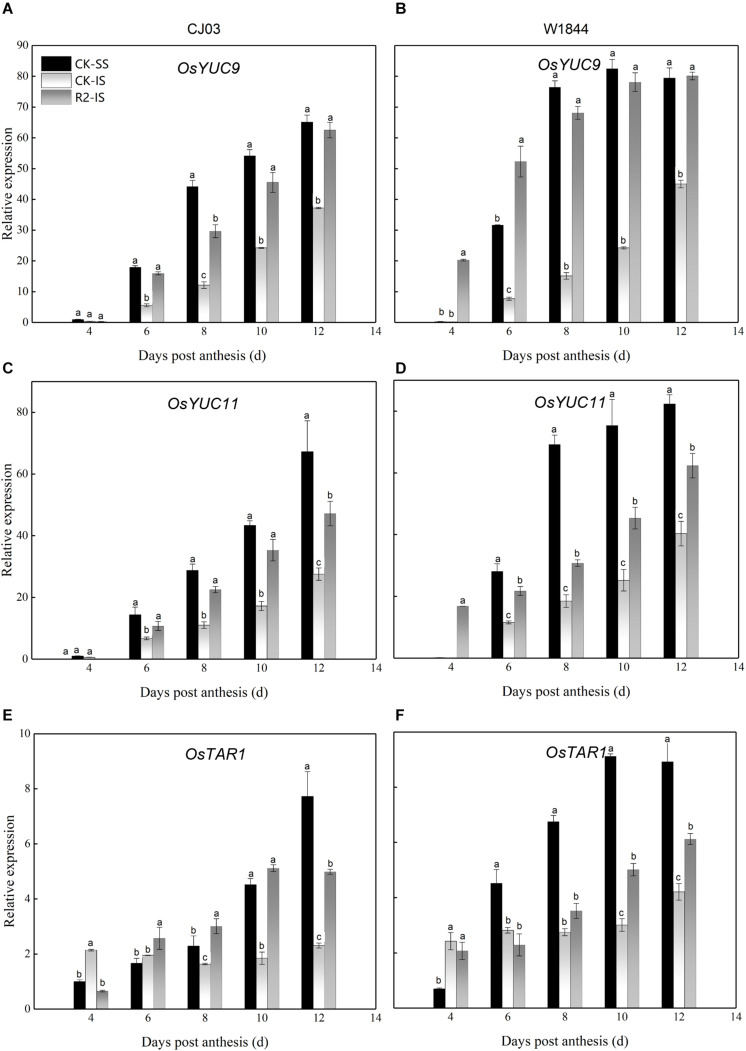
Relative expression levels of *OsYUC9* **(A,B)**, *OsYUC11* **(C,D)**, and *OsTAR1* **(E,F)** in the SS and IS of CJ03 and W1844 at the early grain-filling stage. CK-SS, superior spikelets of control panicle, CK-IS, inferior spikelets of control panicle, R2-IS, inferior spikelets of upper 2/3 removed panicle. Vertical bars represent the mean values ± SE (*n* = 3). Significant differences at each time point are indicated by different letters (*P* < 0.05) as determined by Duncan’s test.

IAA in the developing caryopses likely came from biosynthesis in the leaves or grains (Zhang et al., 2009). We measured the relative expression levels of genes involved in IAA biosynthesis during the initial grain-filling stage (Figure 7). Compared with those in IS, the expression levels of *OsYUC9*, *OsYUC11*, and *OsTAR1* in SS were extremely higher from 6 to 8 DAP. After SS removal, the relative expression levels of these three genes in IS increased rapidly from 6 DAP, especially the relative expression of *OsYUC9* and *OsYUC11* in the IS of W1844, which were at a high level even at 4 DAP.

**FIGURE 8:**
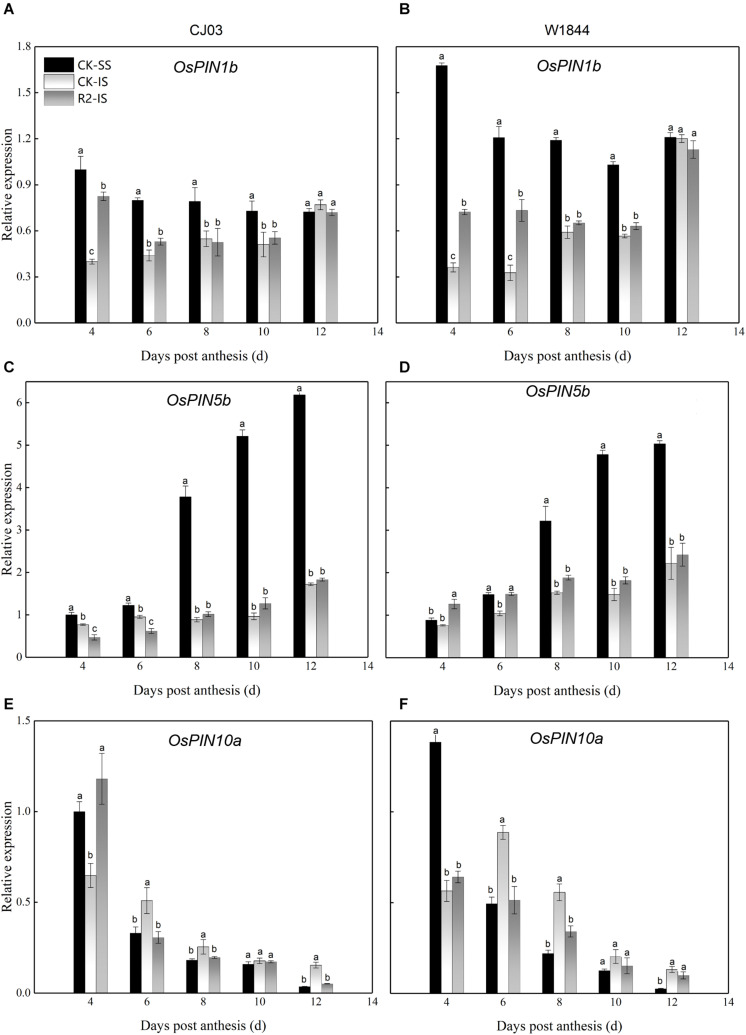
Relative expression levels of *OsPIN1b* **(A,B)**, *OsPIN5b* **(C,D)**, and *OsPIN10a* **(E,F)** in the SS and IS of CJ03 and W1844 at the early grain-filling stage. CK-SS, superior spikelets of control panicle, CK-IS, inferior spikelets of control panicle, R2-IS, inferior spikelets of upper 2/3 removed panicle. Vertical bars represent the mean values ± SE (*n* = 3). Significant differences at each time point are indicated by different letters (*P* < 0.05) as determined by Duncan’s test.

IAA transfers the molecular signal via its polar transport with the help of PIN proteins as auxin efflux carriers (Wang et al., 2009). The results show the different expression patterns of *OsPIN1b*, *OsPIN5b*, and *OsPIN10a* at the initial filing stage (Figure 8). The relative expression levels of *OsPIN1b* and *OsPIN10a* in SS were high at 4 DAP and then decreased, while the expression level of *OsPIN5b* increased after anthesis. At the same time, the expression of these *PIN* genes in IS was always at a lower level. For W1844, the expression levels were higher than those for CJ03 in both SS and IS. After SS removal, the relative expression levels of *OsPIN1b* and *OsPIN10a* for both varieties and *OsPIN5b* for W1844 in IS at 4 DAP increased compared to those in the CK group. Therefore, the high expression of *PIN* genes at the initial stage may be related to the improvements in IS grain filling after spikelet thinning.

### Effects of IAA Application on Inferior Grain Filling

**FIGURE 9:**
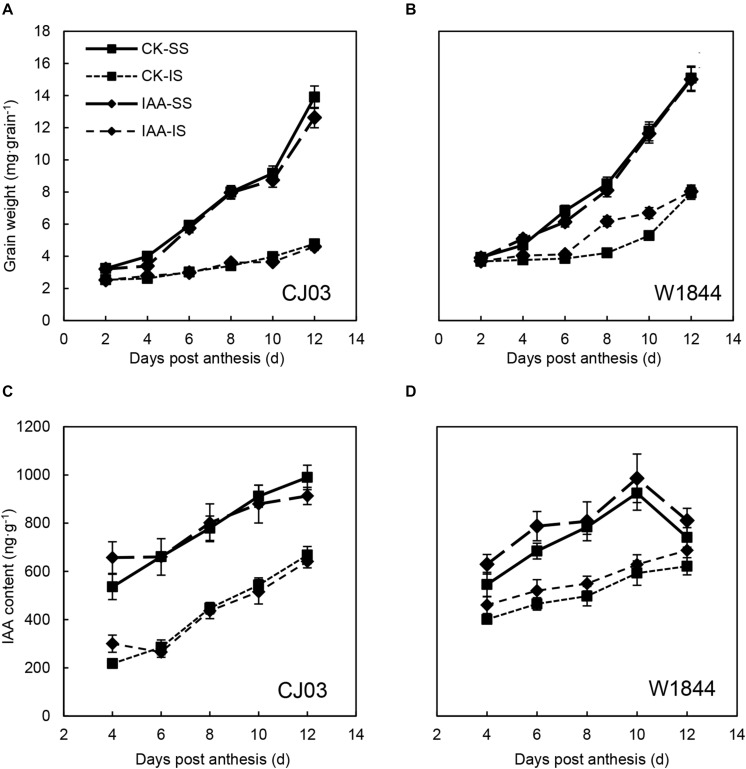
Grain weights **(A,B)** and IAA **(C,D)** contents of the SS and IS of CJ03 and W1844 under IAA application at the early grain-filling stage. CK, control group; IAA, IAA sprayed group. Vertical bars represent the mean values ± SE (*n* = 3).

To verify the roles of IAA in the grain filling of spikelets, IAA was applied at the initial stage of grain filling. As shown in Table 4, IAA application had no significant effects on the final grain weight or seed-setting rate of IS in both varieties. Figure 9 also shows that IAA application did not affect IS grain weight during the initial stage in CJ03, whereas the increasing IS grain weight for W1844 was briefly advanced. After IAA application, the IAA content in the grains was obviously higher than that of the CK group. However, the IAA content in the IS of CJ03 decreased rapidly and returned to a level similar to the CK group, while the IAA content in the IS of W1844 was always at a high level (Figure 9).

**TABLE 4:** Grain weight and seed setting rate of CJ03 and W1844 under IAA application.

Material	Treatment	Grain weight (mg)		Seed settting rate (%)	
		**Superior**	**Inferior**	**Superior**	**Inferior**
W1844	CK	27.50a	22.40c	90.58b	82.15c
	IAA	25.85b	22.30c	94.23a	81.00c
CJ03	CK	25.60a	19.99c	95.74a	83.01b
	IAA	22.97b	19.84c	96.83a	81.37b

*CK, control group; R1, upper part of the spikelets were removed; R2, upper and middle parts of the spikelets were removd; -, the spikelets that were removed. Different lowercase letters following the data that were belong to the same character and variety indicates significant differences at P < 0.05.*

**FIGURE 10:**
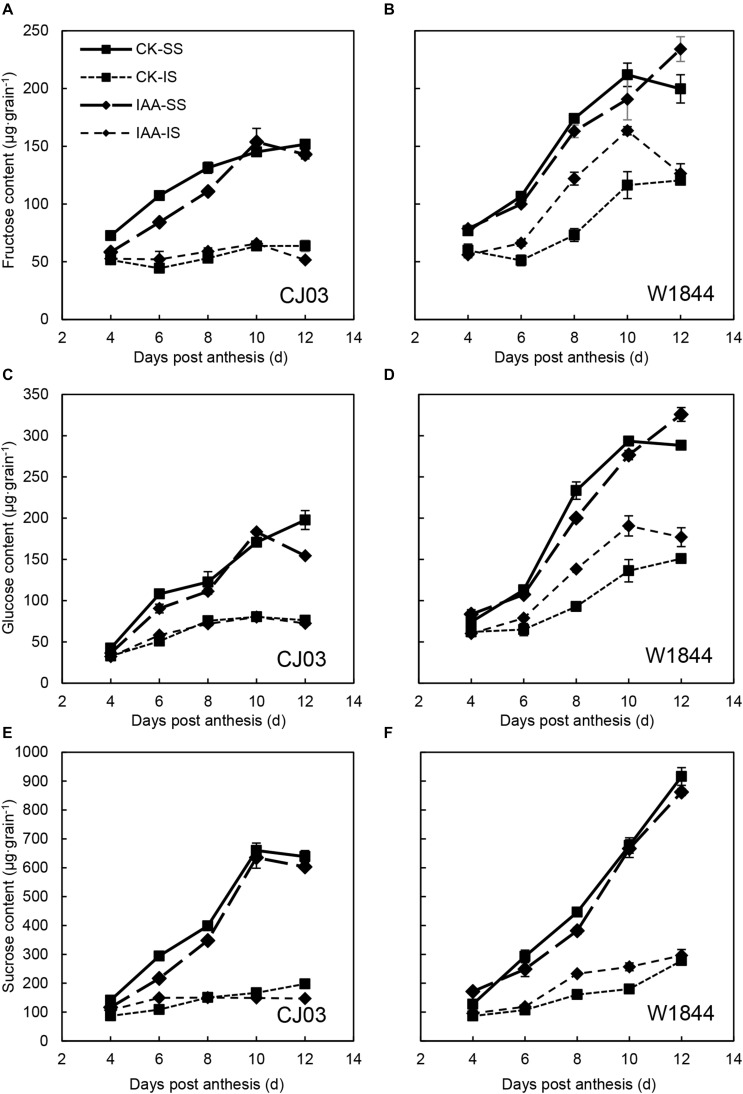
Fructose **(A,B)**, glucose **(C,D)**, and sucrose **(E,F)** contents of the SS and IS of CJ03 and W1844 under IAA application at the early grain-filling stage. CK, control group; IAA, IAA sprayed group. Vertical bars represent the mean values ± SE (*n* = 3).

**FIGURE 11:**
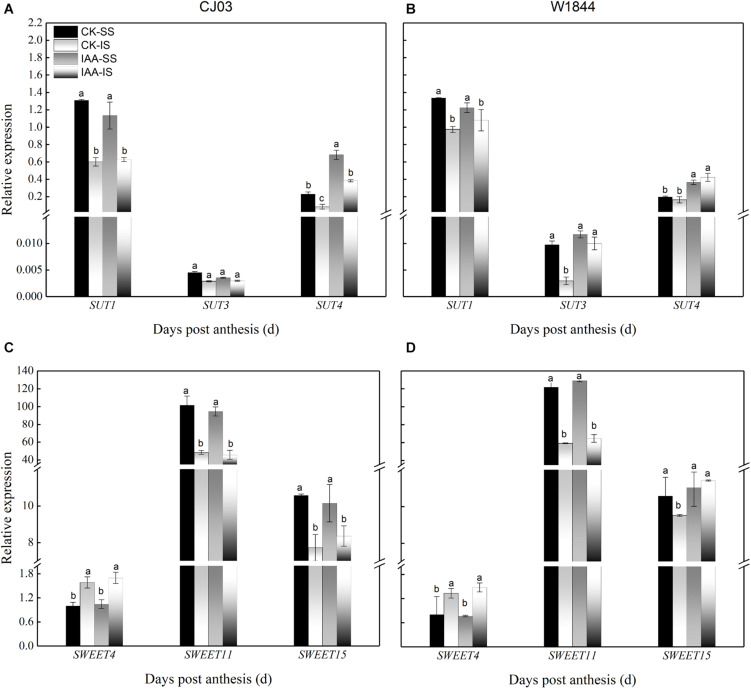
Relative expression levels of *OsSUTs* **(A,B)** and *OsSWEETs* **(C,D)** in the SS and IS of rice under IAA application at 6 DAP. CK, control group; IAA, IAA sprayed group. Vertical bars represent the mean values ± SE (*n* = 3). Significant differences at each time point are indicated by different letters (*P* < 0.05) as determined by Duncan’s test.

Similar to the changes in grain weight, the carbohydrate contents in the IS of W1844 also increased in a short time after IAA application, from 4 to 10 DAP, but the content for CJ03 was not affected (Figure 10). At 6 DAP, the relative expression levels of *OsSUT3*, *OsSUT4*, and *OsSWEET15* in the IS of W1844 increased significantly after IAA application (Figure 11). In summary, exogenous IAA application played a positive role in sucrose transport and accumulation, which resulted in improvements in IS grain filling in W1844.

## Discussion

Poor grain filling of IS is common in large panicle rice (Yang and Zhang, 2006; Ao et al., 2008). There is a 10–15 day developmental stagnancy stage of IS after flowering, which delays the initiation of grain filling and leads to poor filling quality (Zhou et al., 1992; Ishimaru et al., 2003; Zhang et al., 2015). The present study also found that the long lag stage of IS development and grain filling led to asynchronous grain filling between SS and IS in the two large panicle varieties, and the lag time of IS is longer in CJ03 (Chen et al., 2019). Generally, the slow grain filling and low grain weight of IS are considered to be the reasons for sugar limitation (Murty and Murty, 1982; Venkateswarlu and Visperas, 1987; You et al., 2016). The lag time of IS development was shortened after SS removal and was accompanied by the increased sugar content (Figures 1, 2). However, the stagnant stage of IS grain filling did not disappear after spikelet thinning, which indicates the involvement of other factors, such as caryopsis developmental disorders (Ishimaru et al., 2003; Zhang et al., 2009) and poor sucrose translocation (Li et al., 2017; Chen et al., 2019).

The dorsal vascular bundle in the pericarp is the only pathway for sugar to enter the spikelets, and its patency determines the transport efficiency of assimilates (Oparka and Gates, 1984). Transverse section analysis showed that the dorsal phloem cells of the SS were structurally mature shortly after anthesis, with a number of plasmodesmata, while the sieve elements of the IS primarily contained cytoplasm (Figure 3). After SS removal, the development of phloem cells and the patency of the dorsal vascular bundle in the IS improved, and the improvements were more obvious for W1844. These results suggest that the differences between the patency of the dorsal vascular bundle in the SS and IS had effects on the number of carbohydrates entering the grain, which ultimately determined the quality of grain filling.

Before assimilates enter the endosperm, they must traverse a short-distance pathway from the terminal vascular tissue to the aleurone layer (Oparka and Gates, 1981). Because the maternal tissues are isolated from the filial tissues, it is essential to transport the assimilates with the help of transporters by the apoplastic pathway. The relative expression levels of *OsSUT1*, *OsSUT3*, *OsSUT4*, and *OsSWEET11* were always higher in SS than in IS. After SS removal, the expression levels of *OsSUT1*, *OsSUT3*, *OsSUT4*, and *OsSWEET11* in the IS increased, which indicates that the ability of sucrose transport was improved in the IS. After transport to the endosperm, assimilates are soon transformed into starch (Ma et al., 2017). The starch content showed that starch accumulated in a short time and was synthesized by the rich carbohydrates in the endosperm of the IS upon spikelet thinning.

IAA levels are closely associated with grain development, particularly during the initial grain-filling stage. IAA may be the molecular signal for SS inhibition of IS development, and exogenous IAA may reduce this SS inhibition (Tian and Wang, 1998; Wang et al., 2001). The present study demonstrated that the IAA content in SS was much higher than that of IS during initial grain filling and that the IAA content of IS was significantly increased after SS removal. After SS removal, the expression levels of genes involved in IAA biosynthesis (*OsYUC9, OsYUC11*, and *OsTAR1*) and IAA levels increased in the IS, which suggests that the increasing IAA content was primarily from *de novo* synthesis in the grain. Apart from IAA biosynthesis, the expression levels of *PIN* genes coding IAA efflux carriers (*OsPIN1b, OsPIN5b*, and *OsPIN10a*) in IS after spikelet thinning increased shortly after anthesis, which was probably responsible for the development of the dorsal vascular bundle (Scarpella et al., 2010; Zhang et al., 2012). These results indicated that SS removal treatment, which severs the apical dominance of SS, resulted in the improvement of dorsal vascular cell development via IAA polar transport, which allowed the numerous assimilates to enter into the grain and increase IS grain filling. The exogenous IAA application briefly shortened the lag time of IS grain filling for W1844 via increased carbohydrate contents and the expression levels of sucrose transporter genes (*OsSUT3*, *OsSUT4*, and *OsSWEET15*). IAA may be an upstream factor of IS grain filling. SS removal treatment promoted the development of dorsal phloem cells, which allowed numerous assimilates to unload into the grain and be transferred into starch storage in the endosperm, likely mediated via auxin and its polar transport (Figure 10). However, IAA application had no effects on the initiation of IS grain filling for CJ03. After spikelets removal, the IAA content of IS in CJ03 increased, but it is still much lower than its superior spikelets. The IAA content of IS had no significant increase in CJ03 compared to W1844 under IAA application, thus we can see increased grain weight in W1844. The above results demonstrated that CJ03 was tolerant of IAA treatment, and this might due to its lower IAA absorption ability and the different metabolism responses to exogenous IAA. In a future study, more varieties will be used to address the point.

## Data Availability Statement

The original contributions presented in the study are included in the article/supplementary material, further inquiries can be directed to the corresponding author/s.

## Author Contributions

LC, YDe, and YDi designed the experiments. YDe, YY, YH, YL, and LM conducted the experiments. LC, YDe, YW, ZhW, ZiW, and JB analyzed the data and wrote the manuscript. All authors read and approved the final manuscript.

## Conflict of Interest

The authors declare that the research was conducted in the absence of any commercial or financial relationships that could be construed as a potential conflict of interest.
